# Does obesity impact on weaning from invasive ventilation: a secondary analysis of the WEAN SAFE study

**DOI:** 10.1186/s13613-025-01586-1

**Published:** 2025-10-21

**Authors:** Alison Bell, Akira Kuriyama, Omid Khazaei, Bairbre A. McNicholas, Tài Pham, Leo Heunks, Giacomo Bellani, Laurent Brochard, Andrew J. Simpkin, John G. Laffey

**Affiliations:** 1 Department of Anaesthesia and Intensive Care Medicine, Health Services Executive West-Northwest, Galway University Hospitals, Galway, Ireland; 2 Department of Health Research Methods, Evidence, and Impact, McMaster University, Hamilton, Canada; 3 School of Mathematical and Statistical Sciences, University of Galway, Galway, Ireland; 4 Department of Anaesthesia and Intensive Care Medicine, School of Medicine, Clinical Sciences Institute, University of Galway, Galway, H91 YR71 Ireland; 5 Service de Médecine Intensive-Réanimation, AP-HP, Hôpital de Bicêtre, DMU CORREVE, FHU SEPSIS, Groupe de recherche CARMAS, Hôpitaux Universitaires Paris-Saclay, Le Kremlin-Bicêtre, France; 6 Université Paris-Saclay, UVSQ, Univ. Paris-Sud, Inserm U1018, Equipe d’Epidémiologie respiratoire intégrative, CESP, Villejuif, 94807 France; 7 Department of Intensive Care Medicine, Radboud University Medical Centre, Nijmegen, The Netherlands; 8 School of Medicine and Surgery, University of Trento, Trento, Italy; 9 Interdepartmental Division of Critical Care Medicine, University of Toronto, Toronto, Canada; 10 Keenan Research Centre for Biomedical Science, Li Ka Shing Knowledge Institute, St Michael’s Hospital, Unity Health Toronto, Toronto, Canada

**Keywords:** Obesity, Ventilator weaning, Ventilator liberation, Invasive mechanical ventilation

## Abstract

**Objective:**

To understand the impact of obesity on outcomes of weaning from invasive mechanical ventilation (MV).

**Methods:**

The study population consisted of patients enrolled in the WEAN SAFE study. We defined 4 groups based on body mass index (BMI), namely: Normal weight (BMI 18.5–24.9 kg/m²), Overweight (BMI 25–29.9 kg/m²), Obesity Class I (BMI 30–34.9 kg/m²), and obesity classes II and III (BMI ≥ 35 kg/m²). The primary outcome was the rate of successful extubation in patients in each BMI group. Secondary outcomes included the ICU and hospital survival, and PEEP levels at time of weaning eligibility in patients in each BMI group.

**Results:**

In the study population, 1728 (38.2%) were of normal weight, 1395 (30.8%) were overweight, 590 (13.1%) were class I Obesity, and 431 (9.5%) were obesity classes II and III. Patients with obesity were more likely to be female, to be a medical admission, and to have comorbidities. Patients with grade II-III obesity had lower levels of sedation, later timing of the first separation attempt, longer time to weaning success, they received more noninvasive ventilation post extubation, and they had a longer ICU stay. In contrast, weaning success, and ICU and hospital mortality rates were not different in obese patients. There was no independent relationship between obesity and weaning delay, weaning success, or with overall survival outcomes. Higher PEEP at weaning eligibility was associated with weaning failure in normal and overweight patients but not in patients with obesity.

**Conclusions:**

Patients with obesity had a more complex and longer weaning process, but obesity per se was not independently associated with adverse weaning outcomes.

**Supplementary Information:**

The online version contains supplementary material available at 10.1186/s13613-025-01586-1.

## Introduction

Obesity continues to be a growing health concern. In 2022, the World Health Organisation stated that 16% of adults globally are living with obesity (body mass index [BMI] ≥ 30 kg/m²), while 43% are overweight (BMI ≥ 25 kg/m²) [1]. Furthermore, if current trends continue, the Global Burden of Diseases collaborators predict that over half of the global adult population in 2050 will live with overweight or obesity [2].

Obesity is a chronic condition associated with systemic inflammation, metabolic dysfunction, and increased risk of multiple diseases [3]. In intensive care units (ICUs), obesity presents challenges relative to metabolic, respiratory, and hemodynamic management, as well as susceptibility to infections [4]. An increasing number of obese individuals are expected to require critical care in the future [1].

Obesity poses challenges to respiratory mechanics [5–7]. Reductions in respiratory system compliance, functional residual capacity, and expiratory reserve volumes, along with increased airway resistance, contribute to ventilation-perfusion mismatch, and hypoxaemia [5–8]. Increased intra-abdominal and transthoracic pressures can cause diaphragm displacement, leading to atelectasis and impaired gas exchange [9].

These physiological alterations associated with obesity significantly complicate weaning from invasive mechanical ventilation (MV). The intrathoracic pressure theoretically increases with higher BMI, by an estimated 2.5 cmH_2_O for every 10 kg/m² increase in BMI beyond 30 [10]. Consequently, a “one-size-fits-all” approach, where clinicians typically titrate positive end expiratory pressure (PEEP) down to 5 to 10 cmH_2_O during weaning, irrespective of BMI, may increase the risk of atelectasis and subsequently worsen weaning outcomes in patients with obesity. These issues further heighten the risk of ventilator dependence and extubation failure [7].

Delayed and failed weaning from invasive MV is known to worsen patient outcomes, increase mortality risk, and to prolong ICU and hospital stays [11–13]. Our primary aim was to determine the impact of obesity on the weaning process and on outcomes from weaning following invasive MV. Our overall hypothesis was that obesity would prolong the weaning process and reduce weaning success.

## Materials and methods

This is a pre-defined sub-study of the WEAN-SAFE study, an international, multicentre, prospective cohort study of patients undergoing invasive or noninvasive ventilation, conducted during 4 consecutive weeks between October 2017 and June 2018 in a convenience sample of 481 ICUs from 50 countries, across 5 continents, that recruited 5,859 patients that required at least 2 days of invasive MV [12]. The study, jointly supported by the European Society of Intensive Care Medicine (ESICM) and the European Respiratory Society (ERS), was endorsed by multiple national societies/networks (Appendix 1). National coordinators and site investigators (Appendix 1) were responsible for obtaining ethics committee approval and for ensuring data integrity and validity.

### Patients, study design and data collection

All patients aged > 16 years who were admitted to a participating ICU and receiving invasive MV two calendar days after intubation were included in the study. Exclusion criteria included inability to obtain informed consent (where required). Patients transferred to other facilities before successful weaning were deemed lost to follow-up, and their ICU and hospital outcomes were not collected. All data were recorded for each patient at the same time each day within participating ICUs.

### Data definitions

Our data definitions have been previously reported [12]. Briefly, initiation of weaning from invasive MV is defined as the time the first attempt to separate a patient from the ventilator was performed [14]. This ‘separation attempt’ (SA) included spontaneous breathing trials (SBT), i.e. a short period of decreased or absent ventilator support to predict extubation success, or a direct extubation without SBT. For tracheostomized patients, a SA was defined as a short period of either T-tube trial, low respiratory support, a short period of tracheostomy mask oxygenation, or a SBT as declared by the investigator. Weaning eligibility criteria (WEC) were defined as follows: FiO_2_ < 0.5, and positive end-expiratory pressure (PEEP) < 10 cmH_2_O and receiving no or low doses of vasopressors (< 0.2 µg/kg/min of norepinephrine or equivalent), and not receiving paralyzing agents.

As previously reported [12], we used a modified version of the WIND classification [14] to define 5 weaning outcomes:

- “no SA” group: patients who never had a SA in the participating ICU (died or were transferred to another ICU before the first SA).
- “short wean” group: patients were successfully weaned ≤ 1 day after the first SA.
- “intermediate wean” group: patients successfully weaned between 2 and 6 days following the first SA.
- “prolonged wean” group: patients successfully weaned ≥ 7 days after the first SA.
- “failed wean” group: ongoing requirement for invasive ventilatory support at day 90 or at transfer out of the ICU (if sooner), or death (without successful weaning) in patients who underwent a SA.

Delayed weaning initiation is defined as a delay of >1 day from meeting weaning eligibility criteria [12]. Delayed weaning is defined as requiring >1 day to wean from invasive MV following the first SA. Successful weaning is defined as no reintubation within 7 days of extubation. Duration of invasive MV was calculated as the number of days between the date of intubation and the date of extubation in ICU (or death, if the patient died while receiving invasive MV). Survival was evaluated at ICU discharge or at hospital discharge up to a 90-day follow-up. Data about limitation of life sustaining measures was reported.

Sedation was assessed using either the Richmond Agitation Sedation Scale (RASS), the Sedation Agitation Scale (SAS) or the Ramsay Sedation Score (RSS) depending on which scale was in use on the study ICU. The different scales were then aggregated into 3 categories, namely ‘awake’, moderate sedation or deep sedation (Table e1).

We defined 4 study groups based on body mass index (BMI), namely: Normal weight (BMI 18.5–24.9 kg/m²), overweight (BMI 25–29.9 kg/m²), obesity Class I (BMI 30–34.9 kg/m²), and obesity classes II and III (BMI ≥ 35 kg/m²). Underweight patients with a BMI < 18.5 kg/m² (*n* = 221) were excluded from this analysis.

### Outcomes

The primary outcome was the rate of successful extubation in patients in each BMI group. Secondary outcomes included the weaning duration, ICU and hospital survival, end-of-life decision-making in each BMI group. We also examined the risk factors for delayed and failed weaning, and the relationship between PEEP and weaning in patients with obesity.

### Data management and statistical analyses

Descriptive statistics included proportions for categorical and mean (standard deviation) or median (interquartile range) for continuous variables. Differences across BMI categories were assessed using F-tests for categorical variables and Kruskal–Wallis tests for continuous variables.The key exposures in our analysis were the weight categories, with the normal weight group as the comparator. To investigate the association of obesity with weaning duration (0–1 days, 2–6 days, 7 + days) we used an ordinal logistic regression model. First SA, weaning success, ICU mortality, and hospital mortality were time to event outcomes, and were modelled using Cox proportional hazards models. The binary decision to limit life sustaining measures was modelled using a logistic regression model. In each model, we adjusted for age (years), frailty (Clinical Frailty Score >4), gender, admission due to cardiac arrest, trauma or non-traumatic neurological event. We further adjusted for weaning factors, such as PaO_2_/FiO_2_ ratio, dynamic driving pressure, respiratory rate, PEEP, sedation levels on the first day meeting WEC, and whether the patient received paralyzing medication before the first SA. These variables were chosen based on previous research on weaning duration in ICU [12, 15, 16]. An interaction between PEEP and BMI was included in all models to investigate the changing effect of PEEP across BMI categories. For all variables included in the primary and the secondary analysis, we assessed the proportion of missing data (Tables 1 and 2). The overall degree of missingness was low (< 5% for all variables). Accordingly, complete-case analysis was undertaken, as the exclusion of cases with missing covariates was unlikely to introduce substantial bias.

**Table 1:** Demographics and outcome data in patients that entered the weaning process (*n* = 4144 patients)

Characteristic	Normal BMI, *N* = 1,728	Overweight *N* = 1,395	Obesity grade I, *N* = 590	Obesity grade II-III, *N* = 431	Missing Data (%)	*p*-value
Sex: Female	658 (38%)	446 (32%)	246 (42%)	224 (52%)	0 (0%)	< 0.001
Age	59 ± 18	62 ± 16	63 ± 15	60 ± 15	0 (0%)	< 0.001
Frailty: Frail	363 (21%)	253 (18%)	122 (21%)	106 (25%)	6 (0.14%)	0.023
*ICU admission category*						
Medical	1,117 (65%)	910 (65%)	396 (67%)	329 (76.3%)	0 (0%)	0.002
Planned Surgery	164 (9.5%)	115 (8.2%)	47 (8%)	27 (6.3%)	0 (0%)	
Trauma	163 (9.4%)	136 (9.8%)	59 (10%)	22 (5.1%)	0 (0%)	
Urgent Surgery	284 (16.1%)	234 (17%)	88 (15%)	53 (12.3%)	0 (0%)	
*Cause(s) for ICU admission*						
Hypoxemic respiratory failure	541 (31%)	458 (33%)	188 (32%)	183 (42%)	0 (0%)	< 0.001
Hypercapnic respiratory failure	235 (14%)	170 (12%)	91 (15%)	110 (26%)	0 (0%)	< 0.001
Sepsis	349 (20%)	310 (22%)	140 (24%)	122 (28%)	0 (0%)	0.003
Non-traumatic neurologic event	249 (14%)	231 (17%)	64 (11%)	55 (13%)	0 (0%)	0.007
Emergency surgery	263 (15%)	202 (14%)	82 (14%)	47 (11%)	0 (0%)	0.15
Airway protection	215 (12%)	173 (12%)	71 (12%)	56 (13%)	0 (0%)	> 0.9
Cardiac arrest	120 (6.9%)	117 (8.4%)	68 (12%)	38 (8.8%)	0 (0%)	0.006
*Comorbidities and risk factors*						
Respiratory	344 (20%)	265 (19%)	150 (25%)	126 (29%)	0 (0%)	< 0.001
Cardiovascular	203 (12%)	136 (9.7%)	64 (11%)	67 (16%)	0 (0%)	0.009
Liver	73 (4.2%)	58 (4.2%)	27 (4.6%)	21 (4.9%)	0 (0%)	> 0.9
Kidney	194 (11%)	121 (8.7%)	70 (12%)	53 (12%)	0 (0%)	0.037
Neuromuscular	431 (25%)	301 (22%)	119 (20%)	81 (19%)	0 (0%)	0.008
Immune Dysfunction	284 (16%)	175 (13%)	73 (12%)	48 (11%)	0 (0%)	0.002
Diabetes	260 (15%)	296 (21%)	195 (33%)	158 (37%)	0 (0%)	< 0.001
*Outcomes*						
Total duration of invasive mechanical ventilation, days	6 (4, 11)	7 (4, 12)	7 (4, 13)	7 (4, 14)	138 (3%)	< 0.001
Length of ICU stay, days	10 (6, 18)	11 (7, 18)	12 (7, 19)	11 (7, 21)	1 (0.3%)	< 0.001
Length of hospital stay, days	23 (14, 40)	23 (14, 40)	23 (15, 38)	22 (13, 38)	58 (1%)	0.5
Limitation of life-sustaining interventions	275 (16%)	232 (17%)	108 (18%)	79 (18%)	0 (0%)	0.4
ICU mortality, n (%)	213 (13%)	204 (15%)	83 (15%)	68 (16%)	138 (3%)	0.2
Hospital mortality, n (%)	358 (22%)	308 (23%)	128 (22%)	95 (23%)	152 (4%)	0.9

The data are n (%), mean ± SD, or median (IQR).

* p value comparison between the four groups. Kruskal-Wallis for continuous and F-test for categorical

**Table 2:** AHRF severity and ventilatory support data in patients that entered the weaning (*n* = 4144 patients)

Characteristic	Normal BMI, *N* = 1728	Overweight *N* = 1395	Obesity grade I, *N* = 590	Obesity grade II - III, *N* = 431	Missing values	*p*-value
*P/F Ratio*						
At study Inclusion (Day 3–4)	263 ± 104	243 ± 94	230 ± 92	209 ± 87	547 (13%)	< 0.001
First day of fulfilling WEC	288 ± 104	271 ± 92	263 ± 90	243 ± 80	55 (1%)	< 0.001
At first SA	297 ± 104	278 ± 95	271 ± 92	262 ± 98	46 (1%)	< 0.001
*Tidal volumes* (ml/Kg IBW)						
At study Inclusion (Day 3–4)	8.11 ± 2.07	8.13 ± 2.00	8.37 ± 2.22	8.71 ± 2.44	91 (2%)	< 0.001
First day of fulfilling WEC	7.74 ± 1.96	7.73 ± 1.90	8.00 ± 2.10	8.35 ± 2.21	117 (3%)	< 0.001
At first SA	7.72 ± 2.04	7.74 ± 1.97	8.03 ± 2.35	8.34 ± 2.46	104 (2%)	< 0.001
*Respiratory rate*						
At study Inclusion (Day 3–4)	20.5 ± 5.9	20.6 ± 5.7	21.3 ± 5.9	22.0 ± 6.3	19 (0.5%)	< 0.001
First day fulfilling WEC	19.0 ± 5.6	19.3 ± 14.5	19.6 ± 5.7	20.2 ± 6.2	26 (0.6%)	< 0.001
At first SA	20.0 ± 6.0	20.0 ± 6.0	20.0 ± 6.0	21.0 ± 7.0	27 (0.6%)	< 0.001
*PEEP*						
At study Inclusion (Day 3–4)	5 (5, 8)	7 (5, 8)	7 (5, 10)	8 (5, 10)	65 (1%)	< 0.001
First day fulfilling WEC	5 (5, 7)	6 (5, 8)	6 (5, 8)	8 (5, 8)	90 (2%)	< 0.001
At first SA	5 (5, 6)	5 (5, 8)	5 (5, 8)	6 (5, 8)	57 (1%)	< 0.001
*Dynamic compliance (tidal volume/(PIP-PEEP))*						
At study Inclusion (Day 3–4)	0.55 (0.39, 0.80)	0.53 (0.39, 0.75)	0.54 (0.38, 0.76)	0.51 (0.34, 0.74)	202 (5%)	0.005
First day fulfilling WEC	0.59 (0.41, 0.87)	0.57 (0.42, 0.85)	0.60 (0.41, 0.87)	0.58 (0.40, 0.90)	283 (7%)	0.9
At first SA	0.72 (0.49, 1.07)	0.69 (0.48, 1.03)	0.73 (0.50, 1.11)	0.72 (0.51, 1.09)	291 (7%)	0.4
*Dynamic driving pressure (PIP – PEEP)*						
At study Inclusion (Day 3–4)	14 (10, 19)	15 (11, 19)	15 (11, 20)	17 (12, 22)	152 (4%)	< 0.001
First day fulfilling WEC	13 (9, 17)	13 (9, 17)	13 (9, 17)	14 (9, 18)	224 (5%)	0.3
At first SA	10 (7, 15)	11 (8, 15)	11 (7, 15)	11 (8, 15)	237 (6%)	0.2
*Ventilation mode on the first day fulfilling WEC*						
Controlled	540 (32%)	394 (29%)	188 (32%)	138 (33%)	64 (1%)	0.2
Assisted	1,159 (68%)	979 (71%)	396 (68%)	286 (67%)		
*Sedation on the first day fulfilling WEC*						
Awake	413 (24%)	337 (24%)	141 (24%)	123 (29%)	54 (1%)	0.046
Moderate sedation	737 (43%)	592 (43%)	269 (46%)	199 (47%)		
Deep sedation	549 (32%)	451 (33%)	174 (30%)	105 (25%)		
*Adjunctive measures*						
Use of neuromuscular blockade	149 (8.6%)	149 (11%)	59 (10%)	55 (13%)	0 (0%)	0.045

The data are n (%), mean ± SD, or median (IQR).

* p value comparison between the four groups. Kruskal-Wallis for continuous and F-test for categorical

We report the Hazard Ratios (HR) or Odds Ratios (OR) for the Cox and logistic regression models respectively, along with 95% confidence intervals and p-values. All statistical tests were two-tailed, and a *p*-value < 0.05 was considered statistically significant. All analyses were carried out in R software, version 4.4. (R Project for Statistical Computing, http://www.R-project.org).

## Results

**Fig. 1:**
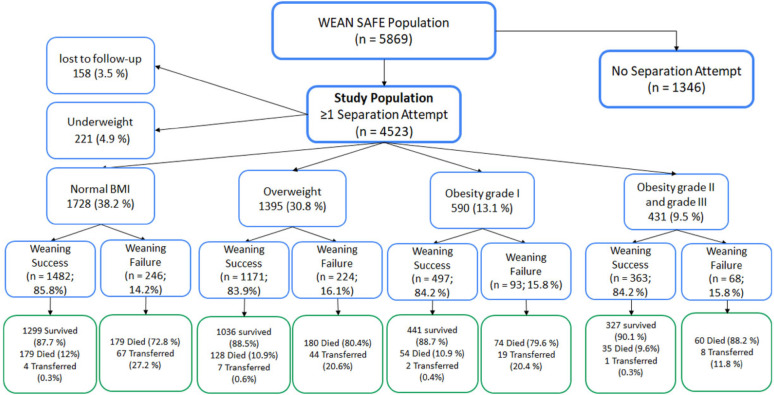
Flow chart for effect of BMI category on the weaning process and outcomes. Of note, patients ‘transferred’ were transferred from the study ICU to another facility while receiving invasive MV, and hence were lost to subsequent followup

Of the 5,869 patients admitted to the participating ICU worldwide that were still receiving invasive ventilation two calendar days after intubation, 4523 (80%) patients entered the weaning process (i.e. underwent a SA), of which 221 were underweight, and were excluded from this analysis. The remaining 4302 patients constitute the study sample (Fig. 1). Of these, 1728 (38.2%) were of normal weight, 1395 (30.8%) were overweight, 590 (13.1%) had class I Obesity, and 431 (9.5%) had class II-III obesity (Fig. 1; Table 1). Patients with obesity were more likely to be female, and to be a medical ICU admission, and to have comorbidities, especially respiratory, cardiovascular or diabetes (Table 1). Patients with grade II-III obesity were more likely to have hypoxaemic or hypercapnic respiratory failure, or sepsis as their cause of ICU admission (Table 1).

### Ventilatory support and respiratory failure severity

Patients with obesity had significantly lower PaO_2_/FiO_2_ ratios at at study inclusion, at first day of fulfilling weaning eligibility criteria (WEC), and at first SA, compared to normal BMI patients (Table 2). Patients with obesity received significantly higher tidal volumes, higher respiratory rates, and higher PEEP levels at study inclusion, at first day of fulfilling weaning eligibility criteria (WEC), and at first SA, compared to normal BMI patients (Table 2).

Patients with obesity had significantly lower dynamic compliance and significantly higher dynamic driving pressure at study inclusion, but not at at first day of fulfilling weaning eligibility criteria (WEC), or at first SA (Table 2). Patients with obesity were more likely to receive neuromuscular blockade compared to other groups (Table 2).

### Initiation of weaning

**Fig. 2:**
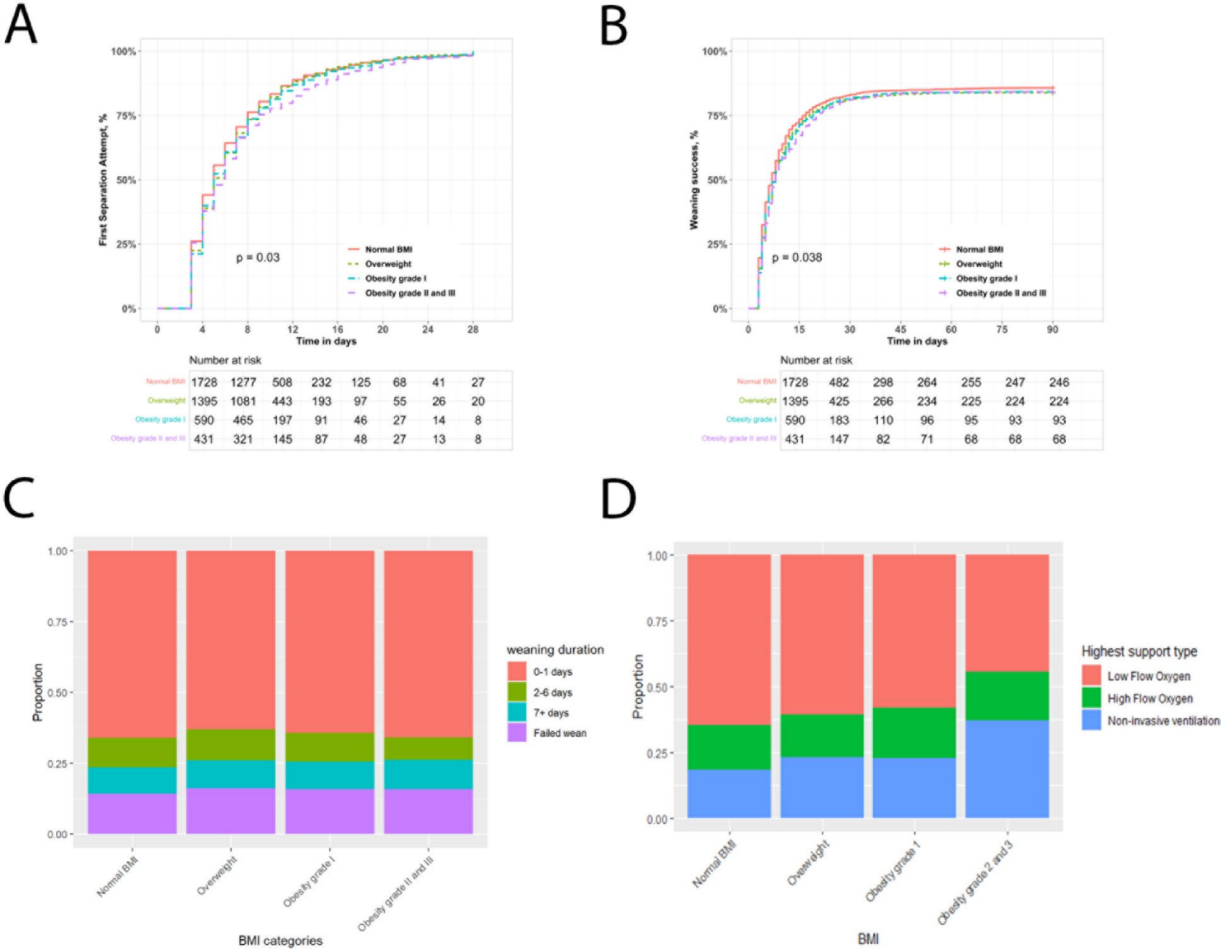
Weaning outcomes in patients with obesity weaning from invasive ventilation. Kaplan-Meier analysis of impact of BMI category on likelihood of entering the weaning process to Day 28 (Panel** A**). Kaplan-Meier analysis of impact of BMI category on weaning success probability over time to Day 90 (Panel** B**). Stacked bar chart of impact of BMI category on weaning outcomes in the study population (Panel** C**). Stacked bar chart of type of respiratory support used post extubation across the BMI category in the study population (Panel** D**)

The frequency of deep sedation at the time of fulfilling WEC was lower, and the frequency of being awake was higher, in patients with class II-III obesity compared to other groups (Table 2). The timing of the first SA was later in patients with grade II-III obesity (Fig. 2A). However, the proportion of patients with delayed weaning from invasive MV was not significantly different across the BMI categories (Table 3). In multivariable analyses, there was no independent effect of BMI group on delayed first SA (Table 4).

**Table 3:** Weaning milestones and outcome data in patients that entered the weaning process (*n* = 4144 patients)

Characteristic	Normal BMI, *N* = 1,728	Overweight *N* = 1,395	Obesity grade I, *N* = 590	Obesity grade II-III, *N* = 431	Missing values (%)	*p*-value
Delayed initiation of weaning	809 (47%)	668 (48%)	281 (48%)	201 (47%)	0 (0%)	> 0.9
*Number separation attempts*						
1	862 (50%)	641 (46%)	272 (46%)	205 (48%)	0 (0%)	0.3
2	484 (28%)	401 (29%)	173 (29%)	118 (27%)		
≥ 3	382 (22%)	353 (25%)	145 (25%)	108 (25%)		
*First separation attempt type*						
Direct Extubation	355 (20%)	294 (21%)	116 (20%)	84 (20%)	0 (0%)	0.7
SBT	1,116 (65%)	915 (66%)	399 (67%)	289 (67%)		
PSV on trach	257 (15%)	186 (13%)	75 (13%)	58 (13%)		
*Number of extubations*						
0	342 (20%)	272 (19%)	104 (18%)	78 (18%)	0 (0%)	0.7
1	1,279 (74%)	1,031 (74%)	440 (75%)	321 (74%)		
≥ 2	107 (6%)	92 (7%)	46 (8%)	32 (8%)		
Reintubation**						
Within 48 h (*n* = 338 patients)	139 (10%)	101 (9%)	50 (10%)	39 (11%)	9 (3%)	0.7
By day 7 (*n* = 479 patients)	195 (14%)	149 (13%)	67 (14%)	57 (16.1%)	11 (2%)	0.6
*Tracheostomy*						
At any time (*n* = 967 patients)	385 (22%)	303 (22%)	118 (20%)	85 (20%)		0.5
Present at Day 3 (*n* = 137 pts)	60 (17%)	41 (14%)	13 (11%)	11 (13%)		0.5
Inserted after at least 1 SA (*n* = 346 patients)	128 (33%)	117 (39%)	43 (36%)	27 (32%)		0.4
*Initial level of post extubation respiratory support*						
Non-Invasive Ventilation	177 (14%)	188 (18%)	75 (17%)	99 (28%)	1057 (26%)	< 0.001
High Flow Oxygen	218 (17%)	165 (16%)	82 (18%)	73 (21%)		
Low flow Oxygen	857 (68%)	682 (66%)	288 (65%)	183 (52%)		
*Highest level of post extubation respiratory support*						
Non-Invasive Ventilation	231 (18%)	241 (23%)	101 (23%)	132 (37%)	1057 (26%)	< 0.001
High Flow Oxygen	210 (17%)	167 (16%)	86 (19%)	66 (19%)		
Low flow Oxygen	811 (65%)	627 (61%)	258 (58%)	157 (44%)		
*Weaning duration (successfully weaned)*						
Short Wean (≤ 1 day)	1,140 (77%)	879 (75%)	379 (76%)	284 (78%)	631 (15%)	0.6
Intermediate Wean (2-7d)	181 (12%)	152 (13%)	60 (12%)	34 (9.4%)		
Prolonged Wean (> 7 days)	161 (11%)	140 (12%)	58 (12%)	45 (12%)		
*Weaning outcomes*						
Failed weaned	246 (14%)	224 (16%)	93 (16%)	68 (16%)	0 (0%)	0.5
Successfully Weaned	1,482 (86%)	1,171 (84%)	497 (84%)	363 (84%)		

The data are n (%).

* p value comparison between the four groups. F-test for categorical.

**Reintubation rates are shown as the proportion of all extubated patients who required reintubation within 48 h and within 7 days, stratified by BMI category. Denominators are the number of patients with 1 or more extubations in each BMI group; patients with missing reintubation status are excluded from denominators

**Table 4:** Multivariable models of association between BMI category on weaning and clinical outcomes

Delayed weaning initiation^1,2^	Adjusted hazard ratio	Lower 95% CI	Upper 95% CI	*p*-value
Overweight	1.006	0.761	1.329	0.967
Obesity grade I	1.022	0.696	1.501	0.913
Obesity grade II and III	1.186	0.772	1.822	0.436

	Adjusted odds ratio	Lower 95% CI	Upper 95% CI	*p*-value
*Weaning duration category* ^1,3^				
Overweight	1.318	0.666	2.607	0.428
Obesity grade I	1.382	0.533	3.578	0.505
Obesity grade II and III	2.260	0.814	6.375	0.120
*Weaning success* ^1,2^				
Overweight	0.860	0.633	1.170	0.337
Obesity grade I	0.712	0.469	1.082	0.112
Obesity grade II and III	0.711	0.451	1.122	0.143
*ICU mortality* ^1,2^				
Overweight	1.18	0.58	2.40	0.64
Obesity grade I	1.64	0.55	4.90	0.38
Obesity grade II and III	0.98	0.30	3.17	0.98
*Hospital mortality* ^1,2^				
Overweight	0.96	0.54	1.71	0.88
Obesity grade I	1.74	0.77	3.91	0.18
Obesity grade II and III	1.18	0.49	2.82	0.71
*Limit life sustaining measures* ^1,4^				
Overweight	0.782	0.373	1.632	0.514
Obesity grade I	1.741	0.612	4.927	0.297
Obesity grade II and III	0.548	0.187	1.566	0.265

^1^Each analysis is adjusted for age, sex, frailty, indices of lung injury severity, sedation at time of fulfilling WEC, reason for ICU Admission, and an interaction between PEEP and BMI category

^2^Multivariable Cox Regression Model

^3^Multivariable Ordinal Logistic regression model

^4^Multivariable Logistic Regression Model

### Weaning events and outcomes

The duration of invasive MV was significantly longer in patients with obesity (Table 1). There was no significant difference in the number of separation attempts, the type of the first separation attempt, the number of extubation attempts, the number of reintubations, or the use of tracheostomy across the weight categories (Table 3).

The time to weaning success to day 90 was longer in patients with grade II-III obesity (Fig. 2B-C), but overall weaning success rates were not different across the weight categories (Table 3). In multivariate analyses, there was no independent effect of BMI group on the duration of weaning, or on weaning success rates (Table 4).

Post extubation, patients with category II-III obesity were more likely to receive noninvasive ventilation, and less likely to receive low flow oxygen, compared to normal BMI patients (Fig. 2D; Table 3).

### Overall clinical outcomes

**Fig. 3:**
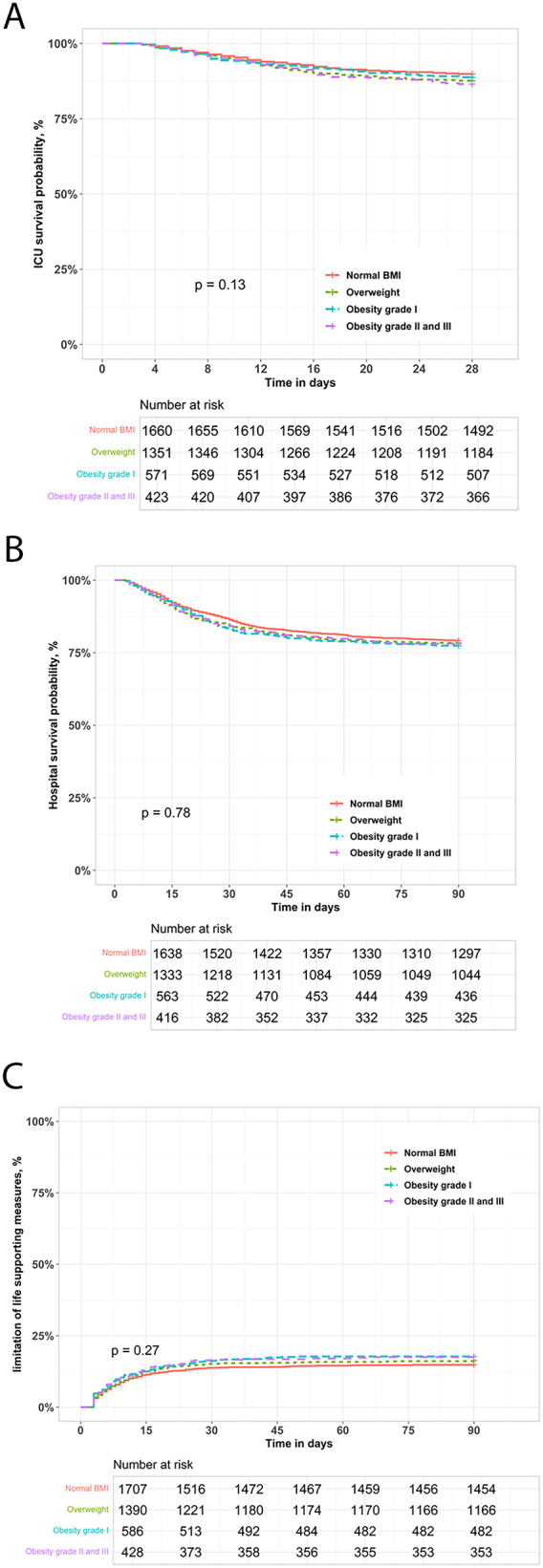
Clincial outcomes in overweight and patients with obesity weaning from invasive ventilation. Kaplan-Meier analysis of ICU survival probability over time to Day 28 (**Panel A**). Kaplan-Meier analysis of hospital survival probability over time to Day 90 (**Panel B**). Kaplan-Meier plot of impact of age/frailty on probability of limitation of life supporting measures (**Panel C**)

The duration of ICU length of stay was significantly longer in patients with obesity (Table 1). In contrast, ICU and hospital survival rates were not significantly different in overweight or patients with obesity, compared to normal-weight patients (Table 1; Fig. 3A-B). Limitation of life-supporting measures was not significantly different between the normal and higher BMI groups (Table 1; Fig. 3C).

In multivariate analyses, there was no independent effect of BMI group on ICU or hospital mortality, or in the frequency of limitation of life supporting measures (Table 4).

### Relationship between PEEP and BMI

**Fig. 4:**
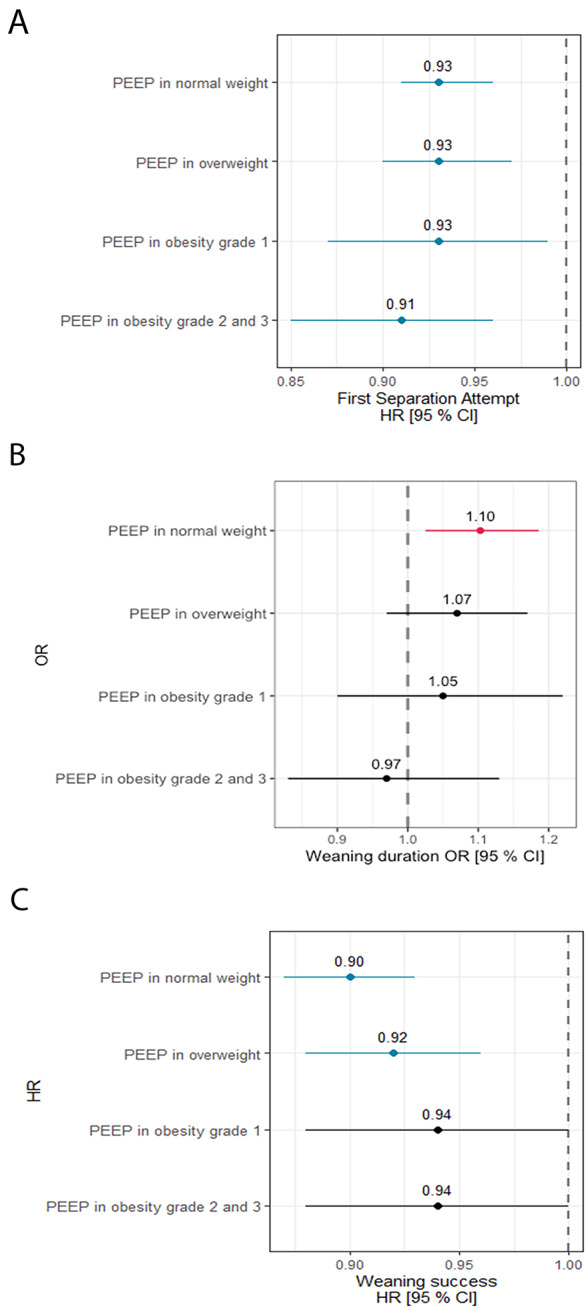
Multivariable analyses of factors associated with the weaning process. Multivariable analysis hazard ratios for factors associated with timing of the first separation attempt (**Panel A**), odds ratios for longer duration of weaning (**Panel B**), and hazard ratios for weaning success (**Panel C**), in patients that entered the weaning process

**Fig. 5:**
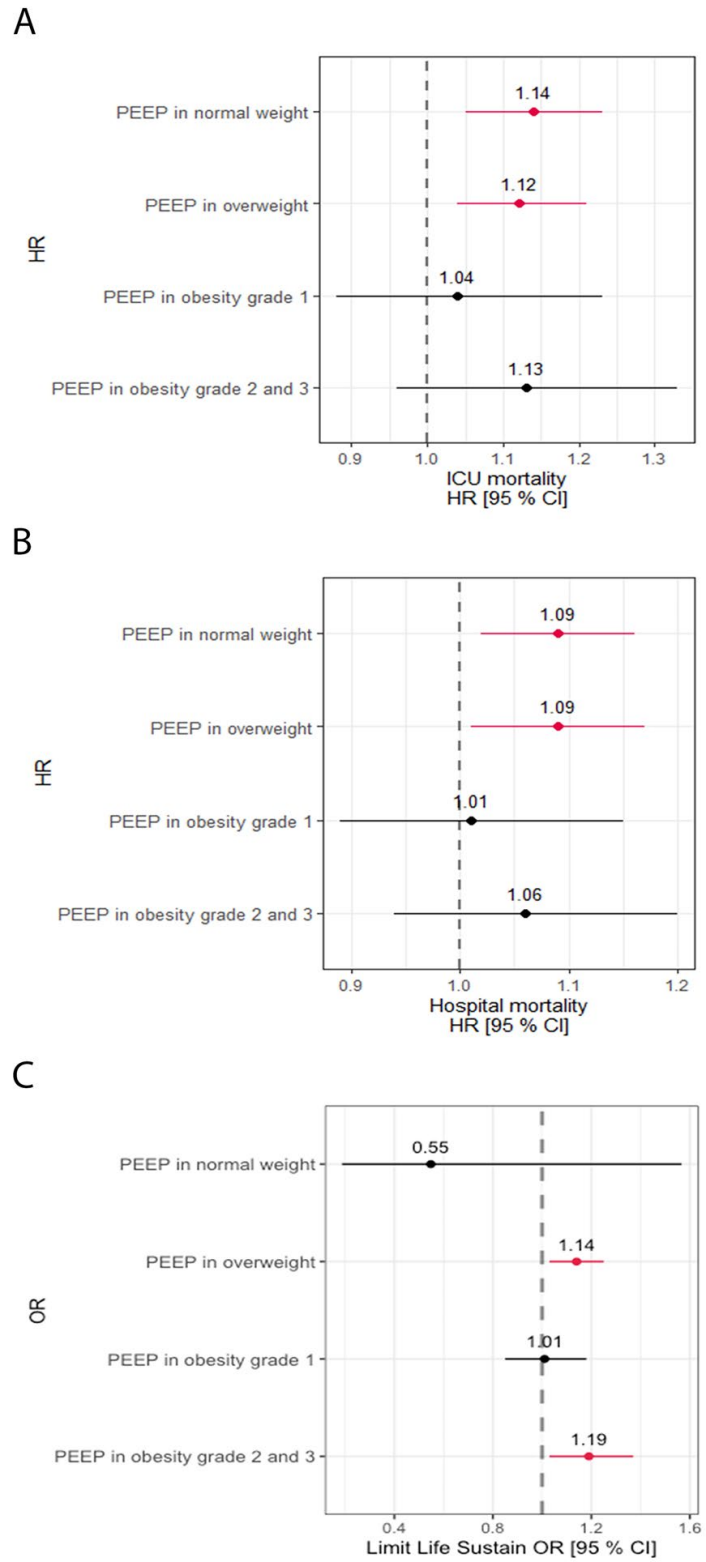
Multivariable analyses of factors associated with weaning process . Multivariable analysis hazard ratios for risk factors for ICU mortality (**Panel A**), hazard ratios for hospital mortality (**Panel B**), and odds ratios for decisions to limit life sustaining measures (**Panel C**), in patients that entered the weaning process

In the whole study sample, higher PEEP at the time of meeting WEC was associated with an earlier first separation attempt, and with increased duration of weaning (Fig. 4A-B, Tables e2 - e3). Of interest, higher PEEP on the first day of meeting WEC was independently associated with higher ICU (Fig. 5A; Table e5) and hospital (Fig. 5B; Table e6) mortality, and with the decision to limit life sustaining measures (Fig. 5C; Table e7). There was no evidence that the effects of PEEP on these outcomes differed signficiantly by BMI group, as there was no evidence for a differential effect of PEEP between different BMI categories (i.e. no significant interaction).

## Discussion

Our findings demonstrate that patients with obesity had a more complex weaning process, but that overall weaning outcomes and survival in patients with obesity were comparable to that seen in the general population. In our analyses, which adjusted for conditions common in patients with obesity, we found no independent relationship between obesity and weaning delays, weaning success rates, or in overall clinical outcomes. This potential for obesity to impact of the weaning process is a concern, given the increasing frequency of obesity in our critically ill patient population, and the central importance of weaning from invasive MV to achieving a favourable outcome from critical illness.

### Impact of obesity on weaning process and outcomes

Patients with class II-III obesity had lower PaO_2_/FiO_2_ ratios, lower dynamic compliance and higher driving pressures and required higher levels of PEEP throughout key stages of the weaning process. Patients with grade II-III obesity had important differences in their weaning process, including lower levels of sedation compared to patients of normal weight. These patients had a later timing of the first separation attempt, and they required longer times to successfully wean from invasive MV. Patients with obesity received more noninvasive ventilation post extubation, and they had a longer ICU stay, supporting previous studies indicating that obesity is associated with greater ventilatory demands [17, 18] and prolonged ICU stays [19–21]. Importantly, overall weaning success rates were comparable across the weight categories.

We found no independent association between BMI category and timing of weaning initiation, weaning duration or ultimate success rates. Our analyses adjusted for factors such as age, frailty status, comorbidities and degree of oxygenation impairment, each factors that are more frequent in patients with obesity. There was no difference in reintubation rates in patients with obesity, with reintubation rates in obese group broadly similar to those previously reported in recent clinical trials [22–24].

Unadjusted ICU and hospital mortality rates, and the use of limitations of life supporting measures were not different in patients with obesity and there was no independent association between BMI category and ICU or hospital survival.

Taken together, these data demonstrate that people with obesity have a more complex and prolonged weaning process and require more post-extubation support and a longer ICU stay. These additional measures during weaning and extubation process appear to be effective in that obesity per se did not have an independent adverse impact on the outcomes of the weaning process, or in ICU or hospital survival, in this cohort.

Our findings contrast with prior studies suggesting an association between obesity and higher failed extubation rates and poorer clinical outcomes [25]. A large-scale US cohort study examining morbidly obese patients from 2004 to 8 found that people with morbid obesity and multiple organ failure were at higher risk of death compared to nonobese people [26] and suggests that advances in care may have improved outcomes in this patient cohort.

Our findings that patients with obesity in the WEAN SAFE cohort had lower levels of sedation when ready to wean, and received more respiratory support post extubation, may explain their better outcomes in this study. Clinicians may have adapted their approach to increase weaning readiness and to pre-empting post-extubation failure in this cohort. The use of noninvasive ventilation has been demonstrated to reduce extubation failure in obese or overweight patients in two recent clinical trials [23, 24], a post hoc review of a clinical trial [22], a network meta-analysis [27] and a retrospective analysis of the FREEREA patient chort [28]. This more pro-active approach may have limited the impact of factors related to obesity such as altered respiratory mechanics, such as decreased chest wall compliance and increased pleural pressure [29], previously suggested to contribute to difficulties in achieving successful liberation from ventilation.

### Relationship between PEEP and obesity in weaning

The relationship between PEEP level at the time of weaning eligibility and outcomes from the weaning process appeared to differ in patients with obesity compared to the non-obese population. In the whole study population, higher PEEP at the time of meeting WEC was associated with worse weaning outcomes, including increased duration of weaning, and reduced weaning success. These poorer weaning outcomes were reflected in a reduced ICU and hospital survival, and increased decisions to limit life supporting measures in patients with higher PEEP requirements. In contrast, in patients with obesity, the relationship between higher PEEP and poorer weaning outcomes and survival rates was lost. Although not statistically significant, the point estimates for risk of ICU and hospital survival were reversed compared to the general population.

Our analysis suggests that, while the probability of weaning initiation was similar per one cm of incremental PEEP on the first day of fulfilling WEC, the first separation attempt was more likely to occur in the obesity class II and III group if on the same level of PEEP. This may imply that patients with the obesity class II and III are more likely to get liberated from invasive MV on a higher PEEP. Obese patients exhibit higher degrees of complete airway closure as reflected by higher opening pressures [30], which may be reduced by higher PEEP levels in these patients. Our finding is supported by a small exploratory study that optimized PEEP using oesophageal balloon measurements to better tailor ventilator settings to obese patients’ respiratory mechanics. While this approach did not change the overall proportion of patients weaned, it did result in faster liberation from mechanical ventilation among those who were successfully weaned [29]. However, whether a high PEEP facilitates the weaning process in patients with obesity remains unclear given the nature of observational study design. Comparatives studies on different PEEP settings in overweight or obese, critically ill patients may be warranted to further examine these issues.

### Strengths and limitations

Our study examines the impact of obesity in patients weaning from invasive MV, performed in a large, and globally diverse, patient cohort. Nevertheless, there are important limitations to consider. While all raw data was entered directly into the electronic case report form, the interpretation of source data was performed by on-site clinicians, which potentially increased variability. To ensure data quality, we instituted a robust data quality control program as previously described [12]. Participating hospitals were representative of different levels of care and geography but despite enrolling a large number of ICUs from around the world, our convenience sample may be prone to selection biases. While adjusted our multivariate analyses for weaning failure for risk factors identified a priori from previous studies [12, 15, 16], additional potential risk factors exist that were not included. Our assumption that patients discharged from the hospital before day 90 were alive at that time point is a further limitation. Lastly, a small proportion of patients (4%) were lost to follow-up because they were transferred prior to the first separation attempt.

## Conclusions

Our study demonstrates that, while patients with obesity had a more complex weaning process, overall weaning outcomes and survival in patients with obesity were comparable to that seen in the general population. Clinicians appear to pay particular attention to these patients, with lower sedation levels, and greater use of noninvasive ventilation post extubation, suggesting an awareness of the greater weaning challenges these patients face. The finding of no independent relationship between obesity and weaning delays, weaning success rates, or in overall clinical outcomes demonstrates that patients with obesity achieve good weaning outcomes despite the pathophysiologic challenges presented.

## Supplementary Information

Below is the link to the electronic supplementary material.

Supplementary Material 1

Supplementary Material 2

## Data Availability

The data in this manuscript are owned by the individual contributing institutions of the WEAN SAFE investigators. Requests for data should be made to the WEAN SAFE Executive Committee, by way of email to the corresponding author. Any data provided will consist of de-identified participant with data dictionary, be restricted to the data presented in this paper, and be subject to a data sharing agreement.
